# Differential Usefulness of Nine Commonly Used Genetic Markers for Identifying *Phytophthora* Species

**DOI:** 10.3389/fmicb.2018.02334

**Published:** 2018-10-03

**Authors:** Xiao Yang, Chuanxue Hong

**Affiliations:** Hampton Roads Agricultural Research and Extension Center, Virginia Tech, Virginia Beach, VA, United States

**Keywords:** oomycetes, plant disease diagnosis, plant pathology, genetics, plant destroyers

## Abstract

The genus *Phytophthora* is agriculturally and ecologically important. As the number of *Phytophthora* species continues to grow, identifying isolates in this genus has become increasingly challenging even by DNA sequencing. This study evaluated nine commonly used genetic markers against 154 formally described and 17 provisionally named *Phytophthora* species. These genetic markers were the cytochrome-*c* oxidase 1 (*cox1*), internal transcribed spacer region (ITS), 60S ribosomal protein L10, beta-tubulin (β*-tub*), elongation factor 1 alpha, enolase, heat shock protein 90, 28S ribosomal DNA, and *tigA* gene fusion protein (*tigA*). As indicated by species distance, *cox1* had the highest genus-wide resolution, followed by ITS, *tigA*, and β*-tub*. Resolution of these four markers also varied with (sub)clade. β*-tub* alone could readily identify all species in clade 1, *cox1* for clade 2, and *tigA* for clades 7 and 8. Two or more genetic markers were required to identify species in other clades. For PCR consistency, ITS (99% PCR success rate) and β*-tub* (96%) were easier to amplify than *cox1* (75%) and *tigA* (71%). Accordingly, it is recommended to take a two-step approach: classifying unknown *Phytophthora* isolates to clade by ITS sequences, as this marker is easy to amplify and its signature sequences are readily available, then identifying to species by one or more of the most informative markers for the respective (sub)clade.

## Introduction

The genus *Phytophthora* currently consists of approximately 200 formal and provisional species with many high-impact plant pathogens (Erwin and Ribeiro, 1996; Yang et al., 2017). For example, *P. infestans* and *P. sojae* are major threats to potato and soybean production, respectively (Erwin and Ribeiro, 1996). *Phytophthora ramorum* (Goheen et al., 2002; Rizzo et al., 2002, 2005) and *P. cinnamomi* (Zentmyer, 1980; Shearer et al., 2004) are destructive forest pathogens causing tree decline in the U.S. and Australia, respectively.

Identifying *Phytophthora* isolates to species is the first and critical step to support plant biosecurity. This process is now done primarily by DNA sequencing. Concerted efforts have been made to identify genetic markers and improve the accuracy of DNA sequence-based identification. As a result, a variety of markers have been identified and utilized (Cooke et al., 2000; Martin and Tooley, 2003; Kroon et al., 2004; Blair et al., 2008; Robideau et al., 2014). Meanwhile, many signature sequences from ex-types (type-derived cultures) and authentic isolates (representative isolates designated by the originators of the respective species) have been generated (Cooke et al., 2000; Kroon et al., 2004; Blair et al., 2008; Martin et al., 2014; Yang et al., 2017), although their availability in public repositories depends upon species (Kang et al., 2010). These two lines of advancement have raised several questions of practical importance. What genetic markers are most useful? Is their resolution dependent upon (sub)clade? How many markers are required to identify *Phytophthora* isolates within a respective (sub)clade to species?

Answers to the above and other related questions will help identifying *Phytophthora* species accurately in the timeliest fashion and at the lowest cost. To this end, Martin et al. (2012) indicated that a set of genetic markers may be required for the most accurate identification. These included the internal transcribed spacer region (ITS), 60S ribosomal protein L10 (60S), beta-tubulin (β*-tub*), elongation factor 1 alpha (*EF-1*α), enolase (ENL), heat shock protein 90 (Hsp90), 28S ribosomal DNA (28S), *tigA* gene fusion protein (*tigA*), cytochrome-*c* oxidase 1 and 2 (*cox1* and *cox2*), subunit 9 of NADH dehydrogenase (*nad9*), ribosomal protein S10 (*rps10*), and SecY protein (*secY*) coding regions. Correspondingly, reference sequences from various markers have been compiled for many known *Phytophthora* species (Cooke et al., 2000; Kroon et al., 2004; Blair et al., 2008; Grünwald et al., 2011; Park et al., 2013; Martin et al., 2014; Yang et al., 2017). In separate studies, Martin et al. (2014) and Martin and Tooley (2003) provided the average pairwise species distances for the concatenated nuclear and mitochondrial genes, and five mitochondrial markers, namely *cox1&2, nad9, rps10*, and *secY*.

The objectives of this study were to evaluate nine commonly used genetic markers against more than 170 *Phytophthora* taxa and identify the most informative markers for individual (sub)clades.

## Materials and methods

### Sequence selection

Nine common genetic markers, namely ITS, *cox1*, 60S, β*-tub, EF-1*α, ENL, Hsp90, 28S, and *tigA*, were evaluated. Sequences of 180 *Phytophthora* isolates representing 154 described and 17 provisionally named species were analyzed. These included 116 ex-types and 28 authentic isolates (Table 1). Eight taxa were represented by two or three isolates due to the lack of sequence data for all regions of individual isolates. The majority of 60S, β*-tub, EF-1*α, ENL, Hsp90, 28S, and *tigA* sequences originated from two previous studies (Blair et al., 2008; Yang et al., 2017). ITS and *cox1* sequences of 90 and 79 *Phytophthora* species, respectively, were downloaded from GenBank (Benson et al., 2018). Sequences from *P*. sp. *ohioensis* (ST18-37) were obtained from the Phytophthora Database (Park et al., 2013). Seventy-nine and 86 isolates were sequenced for ITS and *cox1*, respectively in this study as described below to fill the signature sequence gaps in current public repositories.

**Table 1:** Information and GenBank accession numbers of isolates used in this study.

**(Sub) clade^a^**	**Species**	**Isolate identification**^**b**^					**Type^c^**	**Host or substrate**	**Location**	**Year**	**GenBank accession no**.^**d**^								
		**CH**	**CBS**	**ATCC**	**IMI**	**WPC**					***cox1***	**ITS**	**60S**	*β**-tub***	***EF-1**α*	**ENL**	**Hsp90**	**28S**	***tigA***
1a	*P. cactorum*	22E6				P10194		*Rhododendron* sp.	Ohio, USA	n.a.^e^	MH620014	MH620100	KX250369	KX250370	KX250371	KX250372	KX250373	KX250374	KX250375
	*P. hedraiandra*	62A5	111725			P19523	T	*Viburnum* sp.	The Netherlands	2001	AY769115	AY707987	KX250397	KX250398	KX250399	KX250400	KX250401	KX250402	KX250403
	*P. idaei*	34D4	971.95	MYA-4065	313728	P6767	T	*Rubus idaeus*	Scotland, UK	1987		FJ801946	EU080129	EU080130	EU080131	EU080132	EU080133	EU080134	EU080135
	*P. idaei*				313727		A				AY564185								
	*P. pseudotsugae*			52938	331662	P10339	T	*Psendotsuga menziesii*	Oregon, USA	1975	AY564199	FJ802112	EU080426	EU080427	EU080428	EU080429	EU080430	EU080431	EU080432
1b	*P. clandestina*	32G1	347.86	58713, 60438	278933	P3943	T	*Trifolium subterraneum*	Australia	1985		MH620101	EU079866	EU079867	EU079868	EU079869	EU079870	EU079871	EU079872
	*P. clandestina*				287317						AY564172								
	*P. iranica*	61J4	374.72	60237	158964	P3882	T	*Solanum melongena*	Iran	1969	AY564189	MH620102	KX250439	KX250440	KX250441	KX250442	KX250443	KX250444	KX250445
	*P. tentaculata*	30G8		MYA-3655				*Argyranthemum frutescens*	Germany	2004	MH620015	MH620103	KX250453	KX250454	KX250455	KX250456	KX250457	KX250458	KX250459
1c	*P. andina*					P13365	T	*Solanum brevifolium*	Ecuador	2001		FJ801734	EU080182	EU080183	EU080184	EU080185	EU080186	EU080187	EU080188
	*P. andina*						A				AY564160								
	*P. infestans*	27A8						*Solanum tuberosum*	Mexico	1992	KC733447	KC733443	KX250474	KX250475	KX250476	KX250477	KX250478	KX250479	KX250480
	*P. ipomoeae*	31B5	109229			P10225	T	*Ipomoea longipedunculata*	Mexico	1999	MH620016	MH620104	EU080830	EU080831	EU080832	EU080833	EU080834	EU080835	EU080836
	*P. mirabilis*	30C1		64069, MYA-4062		P3006	A	*Mirabilis jalapa*	Mexico	n.a.	MH620017	MH620105	KX250481	KX250482	KX250483	KX250484	KX250485	KX250486	KX250487
	*P. phaseoli*	23B4						*Phaseolus lunatus*	Delaware, USA	2000	MH620018	MH620106	KX250495	KX250496	KX250497	KX250498	KX250499	KX250500	KX250501
1	*P. nicotianae*	22F9		15410, MYA-4037				*Nicotiana tabacum*	North Carolina, USA	n.a.	KF317091	KF317070	KX250509	KX250510	KX250511	KX250512	KX250513	KX250514	KX250515
2a	*P. botryosa*	62C6	581.69		136915	P3425	T	*Hevea brasiliensis*	Malaysia	1966	MH620019	MH620107	KX250537	KX250538	KX250539	KX250540	KX250541	KX250542	KX250543
	*P. citrophthora*	03E5						Irrigation water	Virginia, USA	2000	KF317096	KF317075	KX250544	KX250545	KX250546	KX250547	KX250548	KX250549	KX250550
	*P. colocasiae*	35D3						*Colocasia esculenta*	Hawaii, USA	2005	KF317097	KF317076	KX250565	KX250566	KX250567	KX250568	KX250569	KX250570	KX250571
	*P. himalsilva*	61G2	128767				T	*Quercus leucotricophora*	Nepal	2005	MH620020	MH620108	KX250572	KX250573	KX250574	KX250575	KX250576	KX250577	KX250578
	*P. meadii*	61J9	219.88		129185			*Hevea brasiliensis*	India	1987	AY564192	MH620109	KX250593	KX250594	KX250595	KX250596	KX250597	KX250598	KX250599
	*P. occultans*	65B9	101557				T	*Buxus sempervirens*	The Netherlands	1998	MH620021	JX978155	KX250600	KX250601	KX250602	KX250603	KX250604	KX250605	KX250606
	*P. terminalis*	65B8	133865				T	*Pachysandra terminalis*	The Netherlands	2010	JX978168	JX978167	KX250607	KX250608	KX250609	KX250610	KX250611	KX250612	KX250613
2b	*P. amaranthi*						T	*Amaranthus tricolor*	Taiwan	2008	n.a.	GU111585	n.a.	KJ179949	n.a.	n.a.	n.a.	n.a.	n.a.
	*P. capsici*	22F4		15399, MYA-4034			A	*Capsicum annum*	New Mexico, USA	1948	KF317094	KF317073	KX250635	KX250636	KX250637	KX250638	KX250639	KX250640	KX250641
	*P. glovera*	62B4	121969			P11685	T	*Nicotiana tabacum*	Brazil	1995	MH620022	MH620110	KX250649	KX250650	KX250651	KX250652	KX250653	KX250654	KX250655
	*P. mengei*	42B2		MYA-4554			T	*Persea americana*	California, USA	n.a.	MH620023	EU748545	KX250656	KX250657	KX250658	KX250659	KX250660	KX250661	KX250662
	*P. mexicana*	45G4	554.88	46731	92550	P0646		*Solanum lycopersicum*	Argentina	n.a.	MH620024	MH620111	KX250670	KX250671	KX250672	KX250673	KX250674	KX250675	KX250676
	*P. siskiyouensis*	41B7	122779	MYA-4187		P15122	T	Stream water	Oregon, USA	2003	KF317102	KF317081	KX250677	KX250678	KX250679	KX250680	KX250681	KX250682	KX250683
	*P. tropicalis*	35C8	434.91	76651, MYA-4218			T	*Macadamia integrifolia*	Hawaii, USA	n.a.	MH620025	MH620112	KX250698	KX250699	KX250700	KX250701	KX250702	KX250703	KX250704
	*P*. sp. brasiliensis			46705		P0630	A	*Theobroma cacao*	Brazil	1969	n.a.	GU259388	EU080419	EU080420	EU080421	EU080422	EU080423	EU080424	EU080425
2c	*P. acerina*	61H1	133931				T	*Acer pseudoplatanus*	Italy	2010	MH620026	JX951285	KX250712	KX250713	KX250714	KX250715	KX250716	KX250717	KX250718
	*P. capensis*	62C1	128319			P1819	T	*Curtisia dentata*	South Africa	n.a.	MH620027	MH620113	KX250726	KX250727	KX250728	KX250729	KX250730	KX250731	KX250732
	*P. citricola*	33H8	221.88	60440	21173	P0716	T	*Citrus sinensis*	Taiwan	1987	KF317095	KF317074	KX250747	KX250748	KX250749	KX250750	KX250751	KX250752	KX250753
	*P. multivora*	55C5	124094				T	Soil	Western Australia, Australia	2007	FJ237508	FJ237521	KX250775	KX250776	KX250777	KX250778	KX250779	KX250780	KX250781
	*P. pachypleura*	61H7			502404		T	*Acuba japonica*	UK	2008	MH620028	MH620114	KX250789	KX250790	KX250791	KX250792	KX250793	KX250794	KX250795
	*P. pini*	45F1		64532			T	*Pinus resinosa*	Minnesota, USA	1925	KF317100	KF317079	KX250810	KX250811	KX250812	KX250813	KX250814	KX250815	KX250816
	*P. plurivora*	22E9		MYA-3657			A	*Kalmia latifolia*	Western Australia, Australia	1998	KF317101	KF317080	KX250817	KX250818	KX250819	KX250820	KX250821	KX250822	KX250823
	*P*. taxon emzansi	61F2					A	*Agathosma betulina*	South Africa	2005	MH620029	MH620115	KX250859	KX250860	KX250861	KX250862	KX250863	KX250864	KX250865
2d	*P. bisheria*	31E6	122081			P10117	T	*Fragaria × ananassa*	North Carolina, USA	1999	MH620030	MH620116	EU080741	EU080742	EU080743	EU080744	EU080745	EU080746	EU080747
	*P. elongata*	55C4	125799				T	Soil	Western Australia, Australia	2004	MH620031	MH620117	KX250894	KX250895	KX250896	KX250897	KX250898	KX250899	KX250900
	*P. frigida*	47G8					T	*Eucalyptus smithi*	South Africa	2001	KF317098	KF317077	KX250915	KX250916	KX250917	KX250918	KX250919	KX250920	KX250921
2e	*P. multivesiculata*	29E3	545.96			P10410	T	*Cymbidium* sp.	The Netherlands	n.a.	MH620032	MH620118	EU080065	EU080066	EU080067	EU080068	EU080069	EU080070	EU080071
	*P*. taxon aquatilis	38J5		MYA-4577			A	Stream water	Virginia, USA	2006	KF317103	FJ666126	KX250929	KX250930	KX250931	KX250932	KX250933	KX250934	KX250935
3	*P. ilicis*	62A7	114348				T	*Ilex aquifolium*	The Netherlands	n.a.	JX524159	JX524158	KX250950	KX250951	KX250952	KX250953	KX250954	KX250955	KX250956
	*P. nemorosa*	41C4		MYA-2948			T	*Lithocarpus densiflorus*	California, USA	n.a.	KF317104	KF317082	KX250964	KX250965	KX250966	KX250967	KX250968	KX250969	KX250970
	*P. pluvialis*	60B3		MYA-4930			T	Rainwater	Oregon, USA	2008	MH620033	MH620119	KX250971	KX250972	KX250973	KX250974	KX250975	KX250976	KX250977
	*P. pseudosyringae*	30A8	111772	MYA-4222			T	*Quercus robur*	Germany	1997	KF317105	KF317083	KX250978	KX250979	KX250980	KX250981	KX250982	KX250983	KX250984
	*P. psychrophila*	29J5	803.95				T	*Quercus robur*	Germany	1995	KF358239	KF358227	KX250992	KX250993	KX250994	KX250995	KX250996	KX250997	KX250998
4	*P. alticola*	47G5	121939			P16948	A	*Eucalyptus dunnii*	South Africa	n.a.	KF317106	KF317084	KX251006	KX251007	KX251008	KX251009	KX251010	KX251011	KX251012
	*P. arenaria*	55C2	127950				T	Soil	Western Australia, Australia	2009	MH620034	MH620120	KX251013	KX251014	KX251015	KX251016	KX251017	KX251018	KX251019
	*P. boodjera*		138637					Soil	Western Australia, Australia	2012	KJ396688	KJ372244	n.a.	KJ372283	n.a.	KJ396738	KJ396710	n.a.	n.a.
	*P. megakarya*	61J5	238.83	42100	202077		T	*Theobroma cacao*	Cameroon	n.a.	MH620035	MH620121	KX251034	KX251035	KX251036	KX251037	KX251038	KX251039	KX251040
	*P. palmivora*	22G9		MYA-4038				*Theobroma cacao*	Costa Rica	n.a.	KF317108	KF317086	KX251055	KX251056	KX251057	KX251058	KX251059	KX251060	KX251061
	*P. quercetorum*	15C7						Soil	South Carolina, USA	1997	KF358240	KF358228	KX251062	KX251063	KX251064	KX251065	KX251066	KX251067	KX251068
	*P. quercina*	30A5	784.95	MYA-4084			T	*Quercus robur*	Germany	1995	KF358241	KF358229	KX252654	KX252655	KX252656	KX252657	KX252658	KX252659	KX252660
	*P*. sp. *ohioensis*					ST18-37	A	Soil	Ohio, USA	2006	n.a.	Phytophthora Database	Phytophthora Database	Phytophthora Database	Phytophthora Database	Phytophthora Database	Phytophthora Database	Phytophthora Database	Phytophthora Database
5	*P. agathidicida*	67D5				P15175	T	*Agathis australis*	New Zealand	2006	MH620036	KP295308	KX251076	KX251077	KX251078	KX251079	KX251080	KX251081	KX251082
	*P. castaneae*	61J7	587.85	36818	325914		T	Soil	Taiwan	n.a.	AY564190	MH620122	KX251097	KX251098	KX251099	KX251100	KX251101	KX251102	KX251103
	*P. cocois*	67D6					T	*Cocos nucifera*	Hawaii, USA	1990	MH620037	KP295304	KX251104	KX251105	KX251106	KX251107	KX251108	KX251109	KX251110
	*P. heveae*	22J1	296.29		180616		T	*Heavae* sp.	Malaysia	n.a.	AY564182	MH620123	KX251111	KX251112	KX251113	KX251114	KX251115	KX251116	KX251117
6a	*P. balyanboodja*		143058					Soil	Western Australia, Australia	2011	MF326863	KJ372258	n.a.	MF326806	n.a.	n.a.	MF326892	n.a.	n.a.
	*P. condilina*		143059					Soil	Western Australia, Australia	2011	MF326843	KJ372262	n.a.	MF326814	n.a.	n.a.	MF326869	n.a.	n.a.
	*P. cooljarloo*		143062					Soil	Western Australia, Australia	2008	HQ012881	HQ012957	n.a.	MF326816	n.a.	n.a.	HQ012925	n.a.	n.a.
	*P. gemini*	46H1	123382				A	*Zostera marina*	The Netherlands	1999	MH620038	FJ217680	KX251125	KX251126	KX251127	KX251128	KX251129	KX251130	KX251131
	*P. humicola*	32F8	200.81	52179, MYA-4080		P3826	T	Soil	Taiwan	1976	KF112862	KF112855	KX251139	KX251140	KX251141	KX251142	KX251143	KX251144	KX251145
	*P. inundata*	30J3			390121		T	*Olea* sp.	Spain	1996	KF112863	KF112856	KX251153	KX251154	KX251155	KX251156	KX251157	KX251158	KX251159
	*P. kwongonina*		143060					Soil	Western Australia, Australia	2010	MF326847	JN547636	n.a.	MF326824	n.a.	n.a.	MF326876	n.a.	n.a.
	*P. pseudorosacearum*		143061					Soil	Western Australia, Australia	2013	MF326858	KJ372267	n.a.	MF326827	n.a.	n.a.	MF326878	n.a.	n.a.
	*P. rosacearum*	47J1		MYA-4456			T	*Malus domestica*	California, USA	n.a.	MH620039	MH620124	KX251445	KX251446	KX251447	KX251448	KX251449	KX251450	KX251451
	*P*. sp. personii					P11555	A	*Nicotiana tabacum*	North Carolina, USA	n.a.		FJ801604	EU080312	EU080313	EU080314	EU080315	EU080316	EU080317	EU080318
	*P*. sp. personii						A				MF326861								
	*P*. taxon walnut	40A7					A	Irrigation water	Virginia, USA	2006	MH620040	MH620125	KX251452	KX251453	KX251454	KX251455	KX251456	KX251457	KX251458
6b	*P. amnicola*	61G6	131652				T	Stream water	Western Australia, Australia	2009	MH620041	MH620126	KX251167	KX251168	KX251169	KX251170	KX251171	KX251172	KX251173
	*P. bilorbang*	61G8	131653				T	Soil	Western Australia, Australia	2010	MH620042	MH620127	KX251181	KX251182	n.a.	KX251183	KX251184	KX251185	KX251186
	*P. borealis*	60B2	132023	MYA-4881			T	Stream water	Alaska, USA	2008	MH620043	MH620128	KX251187	KX251188	KX251189	KX251190	KX251191	KX251192	KX251193
	*P. chlamydospora*			28765	389736		T	*Prunus* sp.	United Kingdom	1971		AF541890		KF750602	n.a.	n.a.			n.a.
						P16851							JF273125					GU594846	
								Soil	Western Australia, Australia	1995	HQ012878						HQ012922		
	*P. crassamura*	66D1	140357				T	Soil	Italy	2012	MH620044	KP863493	KX251201	KX251202	KX251203	KX251204	KX251205	KX251206	KX251207
	*P. fluvialis*	55B6	129424				T	Stream water	Western Australia, Australia	2009	MH620045	MH620129	KX251208	KX251209	KX251210	KX251211	KX251212	KX251213	KX251214
	*P. gibbosa*	62B8	127951				T	Soil	Western Australia, Australia	2009	MH620046	MH620130	KX251222	KX251223	KX251224	KX251225	KX251226	KX251227	KX251228
	*P. gonapodyides*	34A8	554.67	60351		P6872		Reservoir water	n.a.	1967	KC733448	KF112854	KX251236	KX251237	KX251238	KX251239	KX251240	KX251241	KX251242
	*P. gregata*	62B9	127952				T	Soil	Western Australia, Australia	2009	MH620047	MH620131	KX251250	KX251251	KX251252	KX251253	KX251254	KX251255	KX251256
	*P. lacustris*				389725	P10337	T	*Salix matsudana*	England, UK	1972	JF896561	AF266793	EU080530	EU080531	EU080532	EU080533	EU080534	EU080535	EU080536
	*P. litoralis*	55B9	127953				T	Soil	Western Australia, Australia	2008	MH620048	MH620132	KX251278	KX251279	KX251280	KX251281	KX251282	KX251283	KX251284
	*P. megasperma*	62C7	402.72	58817	32035	P3599	T	*Althaea rosea*	Washington DC. USA	1931	MH620049	MH620133	KX251285	KX251286	KX251287	KX251288	KX251289	KX251290	n.a.
	*P. mississippiae*	57J3		MYA-4946			T	Irrigation water	Mississippi, USA	2012	KF112860	KF112852	KX251305	KX251306	KX251307	KX251308	KX251309	KX251310	KX251311
	*P. ornamentata*	66D2	140647				T	Soil	Italy	2012	MH620050	KP863496	KX251319	KX251320	KX251321	KX251322	KX251323	KX251324	KX251325
	*P. pinifolia*	47H1	122924			P16100	T	*Pinus radiata*	Chile	2007	JN935960	MH620134	KX251333	KX251334	KX251335	KX251336	KX251337	KX251338	KX251339
	*P. riparia*	60B1	132024	MYA-4882			T	Stream water	Oregon, USA	2006	MH620051	MH620135	KX251347	KX251348	KX251349	KX251350	KX251351	KX251352	KX251353
	*P. thermophila*	55C1	127954				T	Soil	Western Australia, Australia	2004	MH620052	MH620136	KX251354	KX251355	KX251356	KX251357	KX251358	KX251359	KX251360
	*P. × stagnum*	43F3		MYA-4926			T	Irrigation water	Virginia, USA	2007	KC631619	n.a.	KX251375	KX251376	KX251377	KX251378	KX251379	KX251380	KX251381
6	*P. asparagi*	62C4	132095	MYA-4826			T	*Asparagus officinalis*	Michigan, USA	2006	MH620053	MH620137	KX251473	KX251474	KX251475	KX251476	KX251477	KX251478	KX251479
	*P*. sp. sulawesiensis					P6306	A	*Syzygium aromaticum*	Indonesia	1989	HQ261458	EF590257	EU080345	n.a.	EU080346	EU080347	EU080348	EU080349	EU080350
7a	*P. attenuata*	67C5	141199				T	*Castanopsis carlesii* rhizosphere soil	Taiwan	2013	MH620054	KU517154	KX251609	KX251610	KX251611	KX251612	KX251613	KX251614	KX251615
	*P. europaea*	62A2	109049				T	Soil	France	1998	MH620055	MH620138	KX251522	KX251523	KX251524	KX251525	KX251526	KX251527	KX251528
	*P. flexuosa*	67C3					T	Soil	Taiwan	2013	MH620056	KU517152	KX251616	KX251617	KX251618	KX251619	KX251620	KX251621	KX251622
	*P. formosa*	67C4					T	Soil	Taiwan	2013	MH620057	KU517153	KX251623	KX251624	KX251625	KX251626	KX251627	KX251628	KX251629
	*P. fragariae*	61J3	209.46		181417	P6231	T	*Fragaria × ananassa*	England, UK	n.a.	MH620058	MH620139	KX251543	KX251544	KX251545	KX251546	KX251547	KX251548	KX251549
	*P. intricata*	67B9					T	Soil	Taiwan	2013	MH620059	KU517155	KX251630	KX251631	KX251632	KX251633	KX251634	KX251635	KX251636
	*P. rubi*	46C7		90442			T	*Rubus idaeus* cv. Glen Clova	Scotland, UK	n.a.	DQ674736	HQ643340	KX251564	KX251565	KX251566	KX251567	KX251568	KX251569	KX251570
	*P. uliginosa*	62A3	109054			P10413	T	Soil	Poland	1998	MH620060	MH620140	EU080011	EU080012	EU080013	KX251571	KX251572	EU080015	KX251573
	*P. uniformis*							*Alnus* sp.	Sweden	1996	KU681019	AF139367	n.a.	KU899260	n.a.	n.a.	KU899417	n.a.	n.a.
	*P. × alni*	47A7			392314		T	*Alnus* sp.	UK	1994	KU681017	MH620141	KX251588	KX251589	KX251590	KX251591	KX251592	KX251593	KX251594
	*P. × cambivora*	22F6		46719, MYA-4076				*Abies* sp.	Oregon, USA	n.a.	MH620061	KT183037	KX251494	KX251495	KX251496	KX251497	KX251498	KX251499	KX251500
	*P. × heterohybrida*	67C1	141207				T	Stream water	Taiwan	2013	KU517145	KU517151	KX251637	KX251638	KX251639	KX251640	KX251641	KX251642	KX251643
	*P. × incrassata*	67C2	141209				T	Stream water	Taiwan	2013	KU517150	KU517156	KX251644	KX251645	KX251646	KX251647	KX251648	KX251649	KX251650
	*P. × multiformis*				392316	P16202		*Abies* sp.	The Netherlands	1994	KU681018	AF139368	n.a.	KU899239	n.a.	n.a.	KU899396	n.a.	n.a.
7b	*P. asiatica*	61H3	133347				T	*Pueraria lobata*	Japan	2005	MH620062	MH620142	KX251665	KX251666	KX251667	KX251668	KX251669	KX251670	KX251671
	*P. cajani*	45F7		44388		P3105	T	*Cajanus cajani*	India	n.a.	MH620063	MH620143	KX251686	KX251687	KX251688	KX251689	KX251690	KX251691	KX251692
	*P. melonis*	45F3	582.69	52854			T	*Cucumis sativus*	Japan	n.a.	MH620064	KT183041	KX251707	KX251708	KX251709	KX251710	KX251711	KX251712	KX251713
	*P. niederhauserii*	31E7				P10617	A	*Thuja occidentalis*	North Carolina, USA	2001	MH620065	MH620144	KX251728	KX251729	KX251730	KX251731	KX251732	KX251733	KX251734
	*P. pisi*	60A4					T	Pea	Sweden	2009	MH620066	KT183042	KX251735	KX251736	KX251737	KX251738	KX251739	KX251740	KX251741
	*P. pistaciae*	33D6		MYA-4082	386658		T	*Pistacia vera*	Iran	1986	MH620067	KT183043	KX251748	KX251749	KX251750	KX251751	KX251752	KX251753	KX251754
	*P. sojae*	22D8	312.62	16705, MYA-3899	131375			*Glycine max*	Ontario, Canada	1959	MH620068	MH620145	KX251762	KX251763	KX251764	KX251765	KX251766	KX251767	KX251768
	*P. vignae*	45G6		46735			A	*Glycine max*	n.a.	n.a.	MH620069	MH620146	KX251776	KX251777	KX251778	KX251779	KX251780	KX251781	KX251782
7c	*P. cinnamomi*	61J1	144.22	46671	22938	P2110	T	*Cinnamomum burmannii*	Indonesia	1922	MH620070	MH620147	KX251811	KX251812	KX251813	KX251814	KX251815	KX251816	KX251817
	*P. parvispora*	66C8	132772				T	*Arbutus unedo*	Italy	2011	MH620071	KC478667	KX251839	KX251840	KX251841	KX251842	KX251843	KX251844	KX251845
	*P*. sp. ax	46H5					A	*Ilex glabra* “Shamrock”	Virginia, USA	2008	MH620072	MH620148	KX251846	KX251847	KX251848	KX251849	KX251850	KX251851	KX251852
7d	*P. fragariaefolia*	61H4	135747				T	*Fragaria × ananassa*	Japan	2005	MH620073	MH620149	KX251853	KX251854	KX251855	KX251856	KX251857	KX251858	KX251859
	*P. nagaii*	61H5	133248				T	*Rosa* sp.	Japan	1968	MH620074	MH620150	KX251860	KX251861	KX251862	KX251863	KX251864	KX251865	KX251866
8a	*P. cryptogea*	61H9	113.19		180615	P1738	T	*Solanum lycopersicum*	Ireland	n.a.	MH620075	MH620151	KX251867	KX251868	KX251869	KX251870	KX251871	KX251872	KX251873
	*P. drechsleri*	23J5	292.35	46724		P1087	T	*Beta vulgaris* var. altissima	California, USA	n.a.	MH620076	MH620152	KX251888	KX251889	KX251890	KX251891	KX251892	KX251893	KX251894
	*P. erythroseptica*	61J2	129.23		34684	P1693	T	*Solanum tuberosum*	Ireland	n.a.	MH620077	MH620153	KX251895	KX251896	KX251897	KX251898	KX251899	KX251900	KX251901
	*P. medicaginis*	23A4		MYA-3900				*Medicago sativa*	Ohio, USA	n.a.	KF358236	KF358223	KX251902	KX251903	KX251904	KX251905	KX251906	KX251907	KX251908
	*P. pseudocryptogea*		139749				T	*Isopogon buxifolius*	Western Australia, Australia	2006	KP288342	KP288376							n.a.
	*P. pseudocryptogea*			52402		P3103		*Solanum marginatum*	Ecuador	n.a.			EU080626	EU080627	EU080628	EU080629	EU080630	EU080631	
	*P. richardiae*	45F5	240.3	60353, 46734	325930		T	*Zantedeschia aethiopica*	USA	n.a.	MH620078	MH620154	KX251923	KX251924	KX251925	KX251926	KX251927	KX251928	KX251929
	*P. sansomeana*	47H3		MYA-4455			T	*Glycine* sp.	Indiana, USA	n.a.	MH620079	MH620155	KX251930	KX251931	KX251932	KX251933	KX251934	KX251935	KX251936
	*P. trifolii*	62A9	117687				T	*Trifolium* sp.	Mississippi, USA	n.a.	MH620080	MH620156	KX251958	KX251959	KX251960	KX251961	KX251962	KX251963	KX251964
	*P*. sp. kelmania	24A7		MYA-4162			A	*Abies concolor*	West Virginia, USA	1998	MH620081	MH620157	KX251986	KX251987	KX251988	KX251989	KX251990	KX251991	KX251992
8b	*P. brassicae*	61J8	179.87			P7517, P19521	T	*Brassica oleracea*	The Netherlands	1986	MH620082	MH620158	KX252000	KX252001	KX252002	KX252003	KX252004	KX252005	KX252006
	*P. cichorii*	62A8	115029				T	*Cichorium intybus* var. foliosum	The Netherlands	2004	MH620083	MH620159	KX252007	KX252008	KX252009	KX252010	KX252011	KX252012	KX252013
	*P. dauci*	61E5	127102				T	*Daucus carota*	France	2009	MH620084	MH620160	KX252014	KX252015	KX252016	KX252017	KX252018	KX252019	KX252020
	*P. lactucae*	61F4					T	*Lactuca sativa*	Greece	2001	MH620085	MH620161	KX252042	KX252043	KX252044	KX252045	KX252046	KX252047	KX252048
	*P. primulae*	29E9	620.97					*Primula acaulis*	Germany	1997	KF358238	KF358226	KX252063	KX252064	KX252065	KX252066	KX252067	KX252068	KX252069
	*P. pseudolactucae*		137103					*Lactuca sativa*	Japan	2013	n.a.	AB894388	n.a.	n.a.	n.a.	n.a.	n.a.	n.a.	n.a.
	*P*. taxon castitis	61E7	131246				A	*Fragaria × ananassa*	Sweden	1995	MH620086	MH620162	KX252098	KX252099	KX252100	KX252101	KX252102	KX252103	KX252104
	*P*. taxon parsley	61G1					A	*Petroselinum crispum*	Greece	2006	MH620087	MH620163	KX252105	KX252106	KX252107	KX252108	KX252109	KX252110	KX252111
8c	*P. foliorum*	49J8	121655	MYA-3638		P10974	T	*Rhododendron* sp.	Tennessee, USA	2004	EU124918	MH620164	KX252112	KX252113	KX252114	KX252115	KX252116	KX252117	KX252118
	*P. hibernalis*	22H1	270.31	60352	36906	P6871		*Citrus sinensis*	Portugal	1931	MH620088	KT183039	KX252119	KX252120	KX252121	KX252122	KX252123	KX252124	KX252125
	*P. lateralis*	22H9		MYA-3898			A	*Chamaecyparis lawsoniana*	Oregon, USA	n.a.	MH620089	MH620165	KX252133	KX252134	KX252135	KX252136	KX252137	KX252138	KX252139
	*P. ramorum*	32G2						*Camellia japonica*	South Carolina, USA	n.a.	MH620090	MH620166	KX252147	KX252148	KX252149	KX252150	KX252151	KX252152	KX252153
8d	*P. austrocedrae*	41B6	122911	MYA-4074			T	*Austrocedrus chilensis*	Argentina	2005	KF358233	KF358220	KX252168	KX252169	KX252170	KX252171	KX252172	KX252173	KX252174
	*P. obscura*	60E9	129273				T	Soil	Germany	1994	MH620091	MH620167	KX252175	KX252176	KX252177	KX252178	KX252179	KX252180	KX252181
	*P. syringae*	21H9		34002		P0649		*Citrus* sp.	California, USA	n.a.	MH620092	MH620168	KX252196	KX252197	KX252198	KX252199	KX252200	KX252201	KX252202
8	*P. stricta*	58A1		MYA-4944			T	Irrigation water	Mississippi, USA	2012	KF192702	KF192694	KX252210	KX252211	KX252212	KX252213	KX252214	KX252215	KX252216
9a (cluster 9a1)	*P. aquimorbida*	40A6		MYA-4578			T	Irrigation water	Virginia, USA	2006	GQ294536	FJ666127	KX252238	KX252239	KX252240	KX252241	KX252242	KX252243	KX252244
	*P. chrysanthemi*	61F1	123163				T	*Chrysanthemum × morifolium*	Japan	2000	MH620093	KT183038	KX252266	KX252267	KX252268	KX252269	KX252270	KX252271	KX252272
	*P. hydrogena*	46A3		MYA-4919			T	Irrigation water	Virginia, USA	2007	KC249962	KC249959	KX252280	KX252281	KX252282	KX252283	KX252284	KX252285	KX252286
	*P. hydropathica*	05D1		MYA-4460			T	Irrigation water	Virginia, USA	2000	KC733452	EU583793	KX252294	KX252295	KX252296	KX252297	KX252298	KX252299	KX252300
	*P. irrigata*	23J7		MYA-4457			T	Irrigation water	Virginia, USA	2000	KC733453	EU334634	KX252315	KX252316	KX252317	KX252318	KX252319	KX252320	KX252321
	*P. macilentosa*	58A7		MYA-4945			T	Irrigation water	Mississippi, USA	2012	KF192708	KF192700	KX252343	KX252344	KX252345	KX252346	KX252347	KX252348	KX252349
	*P. parsiana*	47C3			395329		T	*Ficus carica*	Iran	1991	KC733455	KC733446	KX252357	KX252358	KX252359	KX252360	KX252361	KX252362	KX252363
	*P. virginiana*	46A2		MYA-4927			T	Irrigation water	Virginia, USA	2007	KC295546	KC295544	KX252378	KX252379	KX252380	KX252381	KX252382	KX252383	KX252384
	*P*. aff. parsiana G1	47C8					A	*Pistacia vera*	Iran	n.a.	MH620094	MH620169	KX252397	KX252398	KX252399	KX252400	KX252401	KX252402	
	*P*. aff. parsiana G1				395328	P8618		*Pistacia vera*	Iran	1992									EU080201
	*P*. aff. parsiana G2	47C5			395330		A	*Pistacia vera*	Iran	1992	MH620095	MH620170	KX252433	KX252434	KX252435	KX252436	KX252437	KX252438	n.a.
	*P*. aff. parsiana G3	47D8					A	*Pistacia vera*	Iran	n.a.	MH620096	n.a.	KX252463	KX252464	KX252465	KX252466	KX252467	KX252468	n.a.
	*P*. sp. cuyabensis					P8213	A	n.a.	Ecuador	1993	n.a.	FJ802118	EU080664	EU080665	EU080666	EU080667	EU080668	EU080669	EU080331
	*P*. sp. lagoariana	60B5				P8217	T	n.a.	Ecuador	n.a.	MH620097	MH620171	KX252503	KX252504	KX252505	KX252506	KX252507	KX252508	KX252509
9a (cluster 9a2)	*P. macrochlamydospora*-G1	33E1				P10264		*Glycine max*	New South Wales, Australia	n.a.	KC733454	KC733445	KX252510	KX252511	KX252512		KX252513	KX252514	KX252515
	*P. macrochlamydospora*-G1					P10267		*Glycine max*	New South Wales, Australia	1994						EU080007			
	*P. macrochlamydospora*-G2	33D5	240.3	60353	340618			*Zantedeschia aethiopica*	The Netherlands	1927	MH620098	MH620172	KX252516	KX252517	KX252518	n.a.	KX252519	KX252520	KX252521
	*P. quininea*	46C4	407.48	46733			T	*Cinchona officinalis*	Peru	n.a.	MH620099	MH620173	EU079802	EU079803	EU079804	KX252524	EU079805	EU079806	EU079807
9a (cluster 9a3)	*P. insolita*	38E1	691.79	38789	288805		T	Soil	Taiwan	1980	AY564188	GU111612	EU080175	EU080176	EU080177	EU080178	EU080179	EU080180	EU080181
	*P. polonica*	49J9				P15005	A	Soil	Poland	2006	KC733456	KF358225	EU080256	KX252546	EU080258	EU080259	EU080260	EU080261	EU080262
	*P. pseudopolonica*		142610								n.a.	KY707115	n.a.	KY707104	KY787198	n.a.	n.a.	n.a.	n.a.
9b	*P. captiosa*	46H7				P10719	T	*Eucalyptus saligna*	New Zealand	1992	KC733449	MH620174	EU079658	EU079659	EU079660	EU079661	EU079662	EU079663	EU079664
	*P. constricta*	55C3	125801				T	Soil	Western Australia, Australia	2006	KC733450	MH620175	KX252561	KX252562	KX252563	KX252564	KX252565	KX252566	KX252567
	*P. fallax*	46J2				P10722	T	*Eucalyptus delegatensis*	New Zealand	1997	KC733451	MH620176	KX252568	KX252569	KX252570	KX252571	KX252572	KX252573	KX252574
10	*P. boehmeriae*	45F9	291.29		180614	P6950	T	*Boehmeriae nivea*	Taiwan	1927	KT183047	KT183036	EU080161	EU080162	EU080163	EU080164	EU080165	EU080166	EU080167
	*P. gallica*	50A1	111474			P16826	T	*Quercus robur*	France	1998	KF317112	KF317090	KX252589	KX252590	KX252591	KX252592	KX252593	KX252594	KX252595
	*P. gondwanensis*	22G7		MYA-3893				n.a.	Ohio, USA	n.a.	KT183046	KT183035	KX252603	KX252604	KX252605	KX252606	KX252607	KX252608	KX252609
	*P. intercalaris*	45B7	140632	TSD-7			T	Stream water	Virginia, USA	2007	KT163315	KT163268	KX252610	KX252611	KX252612	KX252613	KX252614	KX252615	KX252616
	*P. kernoviae*	46C8				P10956		*Rhododendron ponticum*	England, UK	2004	KT183048	MH620177	EU080041	EU080042	EU080043	EU080044	EU080045	EU080046	KX252631
	*P. morindae*	62B5	121982				T	*Morinda citrifolia* var. citrifolia	Hawaii, USA	2005	KT183050	MH620178	KX252633	KX252634	KX252635	KX252636	KX252637	KX252638	KX252639
	*P*. sp. boehmeriae-like	45F8	357.52	60173	32199	P1378	A	*Citrus sinensis*	Argentina	1939	KF317111	KF317089	KX252640	KX252641	KX252642	KX252643	KX252644	KX252645	KX252646
n.a.	*P. lilii*		135746				T	*Lilium* sp.	Japan	1987	AB856786	MG865523	AB856779	AB856782	AB856788	AB856791	AB856794	AB856797	AB856800
Outgroup	*Elongisporangium undulatum*		101728		337230	P10342	T	*Larix* sp.	Scotland, UK	1989		FJ802126		EU080441					EU080445
	*Pythium aphanidermatum*										AY564163								

a *Molecular phylogenetic (sub)clade as indicated by the concatenated-sequence tree (TreeBASE S22998)*.

b *Isolate identification: CH, Chuanxue Hong laboratory at Virginia Polytechnic Institute and State University, Virginia Beach, VA, USA; CBS, Centraalbureau voor Schimmelcultures Fungal Biodiversity Centre, Utrecht, The Netherlands; ATCC, American Type Culture Collection, Manassas, VA, USA; IMI, CABI Biosciences, UK; WPC, the World Phytophthora Genetic Resource Collection at University of California, Riverside, USA*.

c *Ex-types (T) or authentic (A) isolates (designated as representative isolates by the originators of the respective species)*.

d *Marker: cox1, cytochrome-c oxidase 1 gene; ITS, internal transcribed spacer region; 60S, 60S Ribosomal protein L10; β-tub, beta-tubulin; EF-1α, elongation factor 1 alpha; ENL, enolase; Hsp90, heat shock protein 90; 28S, 28S ribosomal DNA; tigA, tigA gene fusion protein*.

e *n.a., not available*.

### DNA extraction, amplification, and sequencing

To extract genomic DNA (gDNA), a 5 × 5 mm agar plug was cut from the actively growing edge of a fresh culture and transferred to 20% clarified V8 broth. Cultures were incubated at room temperature (*c*. 23°C) for 7–14 d to produce a mycelial mass. The mass was blotted dry on sterile tissue paper, transferred to a garnet bead tube and lysed in a FastPrep®-24 (MP Biomedicals, Santa Ana, CA). gDNA was purified using a custom Maxwell® 16 FFS nucleic acid extraction kit in combination with a Maxwell® Rapid Sample Concentrator (Promega, Madison, WI).

A pair of primers including the forward primer ITS6 and reverse primer ITS4 (Cooke et al., 2000) was used to amplify the ITS region. The *cox1* fragment was amplified with the primer pair COXF4N and COXR4N (Kroon et al., 2004). PCR reaction mixtures were prepared with Takara *Taq* DNA polymerase (Takara Shuzo, Shiga, Japan) according to the manufacturer's instructions. Each *cox1* PCR reaction mixture contained an additional 2-μL 25 mM MgCl_2_ and 0.25-μL Bovine serum albumin (BSA) per 25-μL. Thermal cycling protocols were described previously (Cooke et al., 2000; Kroon et al., 2004). All PCR products were evaluated for successful amplification using agarose gel electrophoresis. Sequencing reactions were run in both directions with the same primer pairs used for amplification at the University of Kentucky Advanced Genetic Technologies Center (Lexington, KY) or Eton Bioscience Inc. (Durham, NC). Results were viewed in Finch TV version 1.4.0 (Geospiza, Seattle, WA), aligned using Clustal X (Larkin et al., 2007), and edited manually to correct obvious sequencing errors and code ambiguous sites according to the International Union of Pure and Applied Chemistry (IUPAC) nucleotide ambiguity codes to produce a consensus sequence. All sequences produced in this study have been deposited in GenBank (Table 1).

Rates of PCR success for all nine genetic markers were estimated by calculating the percentage of successful amplifications over all PCR reactions performed by the authors for each marker during the past 6 years.

### Genus-wide distance analyses

All nine genetic markers were analyzed for overall species distances resolved across the genus *Phytophthora*. Sequence datasets of each marker were aligned using the MUSCLE version 3.7 (Edgar, 2004) in MEGA version 7.0.26 (Kumar et al., 2016). Alignments were manually modified when obvious errors were present. The alignment of each marker was then trimmed to an equal size and question marks were inserted to represent missing data at both ends of short sequences. DNA sequence distances were calculated using the Kimura 2-parameter (K2P) distance model (Kimura, 1980) to explore the maximum, minimum and mean distances across the genus.

### Distance analyses within individual (sub)clades

Four selected markers that had relatively high mean species distances across the genus (*cox1*, ITS, *tigA*, and β*-tub*) were analyzed for distances within individual (sub)clades. Phylogenetic (sub)clade assignments for each species were identified according to the recent study by Yang et al. (2017). Sequence datasets within individual (sub)clades of each marker were aligned and edited as described above. Maximum, minimum, and mean distances within individual (sub)clades of each marker were calculated as described above.

### Comparison of individual-marker trees with concatenated-sequence tree

Each marker tree for all four selected markers (*cox1*, ITS, *tigA*, and β*-tub*) included a set of identical 150 *Phytophthora* taxa, plus two outgroup taxa: *Elongisporangium undulatum* (basionym: *Pythium undulatum*) was used as the outgroup taxon for ITS, *tigA*, and β*-tub*, while *Pythium aphanidermatum* (Uzuhashi et al., 2010) was used for the mitochondrial marker *cox1*. The sequence dataset of each marker was aligned in MEGA 7 and edited as described above. Then, the four alignments were combined in MEGA 7 to produce a concatenated sequence alignment. Phylogeny reconstructions including four individual-marker trees and a concatenated-sequence tree were carried out using both Maximum likelihood (ML) and Neighbor joining (NJ) methods with the K2P model and 1000 bootstrap replications in MEGA 7. Alignments and phylogenetic trees have been deposited in TreeBASE (S22998).

To validate the accuracy of the concatenated-sequence trees, the clade affiliation of individual species was compared with those presented in previous phylogenetic studies. The overall topological scores between the concatenated-sequence trees and individual-marker trees were calculated using Compare2Trees version September 2011 (Nye et al., 2006).

## Results

### PCR consistency, amplifications, and sequence alignments

The ITS region was the easiest genetic marker to amplify (Table 2). The rates of PCR amplification success for β*-tub*, 28S, 60S, Hsp90, and *EF-1*α were also high (>90%; Table 2). Markers *tigA*, ENL, and *cox1* (using primer pair COXF4N and COXR4N) had relatively low success rates (≤ 80%) with the *tigA* being the most difficult (Table 2).

**Table 2:** PCR consistency and overall species distance across the genus *Phytophthora* by genetic marker.

**Marker^a^**	**Internal primers used^b^**	**Aligned length (bases)**	**No. of amplifications**	**No. of species^d^**	**Rate of PCR success (%)^c^**	**Mean distance^e^**	**Distance range**
*cox1*	No	867	456	165	75	0.092 ± 0.0003	0–0.256
ITS	No	1,039	408	169	99	0.082 ± 0.0003	0–0.182
*tigA*	Yes	1,670	284	155	71	0.058 ± 0.0001	0–0.110
*β-tub*	No	1,136	502	169	96	0.043 ± 0.0001	0–0.087
ENL	No	1,168	345	159	80	0.035 ± 0.0001	0–0.097
28S	No	1,273	323	160	97	0.035 ± 0.0002	0–0.088
60S	No	496	344	160	97	0.030 ± 0.0001	0–0.085
Hsp90	Yes	1,758	330	168	95	0.024 ± 0.0001	0–0.116
*EF-1α*	No	1,015	299	159	98	0.008 ± 0.0000	0–0.024

a *Marker: cox1, cytochrome-c oxidase 1 gene; ITS, internal transcribed spacer region; 60S, 60S Ribosomal protein L10; β-tub, beta-tubulin; EF-1α, elongation factor 1 alpha; ENL, enolase; Hsp90, heat shock protein 90; 28S, 28S ribosomal DNA; tigA, tigA gene fusion protein*.

b *Internal primers for sequencing tigA and Hsp90 are listed in Blair et al. (2008)*.

c *Rate of successful PCR amplification for each marker done by the authors during the past 6 years*.

d *Number of species (one isolate per species) included in the sequence alignment of each marker*.

e *Overall species distance (mean ± standard error) calculated using the Kimura 2-parameter (K2P) distance model in MEGA 7*.

Sequences could not be obtained from 11 taxa for 60S, 2 for β*-tub*, 12 for *EF-1*α, 12 for ENL, 3 for Hsp90, 11 for 28S, 16 for *tigA*, 2 for ITS, and 6 for *cox1* (Table 2). These taxa were excluded from distance analyses of individual markers. Eleven taxa missing any of *cox1*, ITS, *tigA*, or β*-tub* sequences were also excluded in the comparison of individual-marker trees with concatenated-sequence tree.

Sequence lengths were consistent for all markers except for the ITS, in spite of missing data at either or both ends of short sequences. Length of ITS sequences varied from 744 bases (*P. hydrogena* in cluster 9a1 of subclade 9a) to 848 bases (*P. intercalaris* in clade 10).

Among the nine markers, the aligned length was the shortest for 60S and longest for Hsp90 (Table 2). The aligned length of concatenated sequences (*cox1*, ITS, *tigA*, and β*-tub*) of 150 *Phytophthora* taxa plus the outgroup was 4,714 bases.

### Genus-wide distance analyses

The mean species distance of *cox1* was the highest among the nine markers (Table 2). ITS had the highest genus-wide resolution among the nuclear markers, followed by *tigA* and β*-tub* (Table 2). ENL, 28S, 60S, Hsp90, and *EF-1*α had lower species distances (mean distance < 0.04). *EF-1*α had the lowest resolution across the genus (Table 2). Species pairs with identical sequences (distance = 0) were found for all markers.

### Distances within individual (sub)clades

Four markers including *cox1*, ITS, *tigA*, and β*-tub*, were selected for distance analyses within individual (sub)clades. Species distances (mean values and ranges) for 10 Phytophthora clades and 20 subclades according to previously assigned numbers (Yang et al., 2017) are listed in Table 3.

**Table 3:** Species distance of the four most informative genetic markers with recommendations for identifying isolates in each Phytophthora (sub)clade.

**Clade**		**Marker**				**Recommended markers for unambiguous ID**
		***cox1***	**ITS**	***tigA***	*β**-tub***	
1	1a	0.008–0.026 (0.018_mean_ ± 0.002_se_)	0.004–0.009 (0.006_mean_ ± 0.001_se_)	0.008–0.024 (0.018_mean_ ± 0.003_se_)	0.003–0.004 (0.004_mean_ ± 0.0003_se_)	All
	1b	0.003–0.073 (0.049_mean_ ± 0.023_se_)	0.014–0.027 (0.022_mean_ ± 0.004_se_)	0.013–0.061 (0.041_mean_ ± 0.014_se_)	0.028–0.035 (0.031_mean_ ± 0.002_se_)	All
	1c	0.008–0.019 (0.013_mean_ ± 0.001_se_)	0–0.003 (0.001_mean_ ± 0.0003_se_)	0–0.009 (0.005_mean_ ± 0.001_se_)	0.002–0.007 (0.004_mean_ ± 0.001_se_)	*cox1* and *β-tub*
	**Overall**	**0–0.075 (0.047**_mean_ ±**0.003**_se_**)**	**0–0.070 (0.047**_mean_ ±**0.003**_se_**)**	**0–0.108 (0.072**_mean_ ±**0.004**_se_**)**	**0.002–0.077 (0.046**_mean_ ±**0.003**_se_**)**	*β**-tub***
2	2a	0.003–0.082 (0.040_mean_ ± 0.006_se_)	0.001–0.010 (0.005_mean_ ± 0.001_se_)	0.001–0.028 (0.017_mean_ ± 0.002_se_)	0–0.026 (0.012_mean_ ± 0.002_se_)	*cox1*
	2b	0.004–0.039 (0.024_mean_ ± 0.003_se_)	0.004–0.019 (0.012_mean_ ± 0.001_se_)	0.006–0.044 (0.034_mean_ ± 0.003_se_)	0–0.037 (0.021_mean_ ± 0.002_se_)	*cox1*, ITS, and *tigA*
	2c	0.011–0.051 (0.027_mean_ ± 0.002_se_)	0.003–0.022 (0.009_mean_ ± 0.001_se_)	0.004–0.046 (0.024_mean_ ± 0.003_se_)	0.001–0.027 (0.012_mean_ ± 0.001_se_)	*cox1*, ITS, and *tigA*
	2d	0.029–0.038 (0.035_mean_ ± 0.003_se_)	0.028–0.056 (0.041_mean_ ± 0.008_se_)	0.066–0.095 (0.082_mean_ ± 0.008_se_)	0.023–0.043 (0.035_mean_ ± 0.006_se_)	All
	2e	0.027	0.018	0.023	0.012	All
	**Overall**	**0.003–0.089 (0.045**_mean_ ±**0.001**_se_**)**	**0–0.085 (0.032**_mean_ ±**0.001**_se_**)**	**0.001–0.135 (0.073**_mean_ ±**0.002**_se_**)**	**0–0.081 (0.043**_mean_ ±**0.001**_se_**)**	***cox1***
3		**0.027–0.058 (0.046**_mean_ ±**0.004**_se_**)**	**0.001–0.010 (0.006**_mean_ ±**0.001**_se_**)**	**0.012–0.022 (0.016**_mean_ ±**0.001**_se_**)**	**0.004–0.014 (0.009**_mean_ ±**0.001**_se_**)**	***cox1***, ***tigA*****, and** *β**-tub***
4		**0.004–0.064 (0.036**_mean_ ±**0.004**_se_**)**	**0.001–0.111 (0.061**_mean_ ±**0.007**_se_**)**	**0.005–0.135 (0.095**_mean_ ±**0.009**_se_**)**	**0.002–0.076 (0.047**_mean_ ±**0.005**_se_**)**	***cox1***, ***tigA*****, and** *β**-tub***
5		**0.008–0.037 (0.023**_mean_ ±**0.006**_se_**)**	**0–0.012 (0.006**_mean_ ±**0.002**_se_**)**	**0.003–0.011 (0.007**_mean_ ±**0.001**_se_**)**	**0.002–0.006 (0.004**_mean_ ±**0.001**_se_**)**	***cox1***, ***tigA*****, and** *β**-tub***
6	6a	0.010–0.056 (0.036_mean_ ± 0.002_se_)	0.001–0.066 (0.029_mean_ ± 0.002_se_)	0.004–0.086 (0.055_mean_ ± 0.005_se_)	0–0.052 (0.031_mean_ ± 0.002_se_)	*cox1* and *tigA*
	6b	0.015–0.077 (0.053_mean_ ± 0.001_se_)	0–0.040 (0.021_mean_ ± 0.001_se_)	0.015–0.039 (0.025_mean_ ± 0.0004_se_)	0.001–0.031 (0.018_mean_ ± 0.001_se_)	*cox1* and *tigA*
	**Overall**	**0.010–0.116 (0.064**_mean_ ±**0.001**_se_**)**	**0–0.098 (0.036**_mean_ ±**0.001**_se_**)**	**0.003–0.111 (0.042**_mean_ ±**0.001**_se_**)**	**0–0.054 (0.025**_mean_ ±**0.001**_se_**)**	***cox1*****and** ***tigA***
7	7a	0–0.042 (0.021_mean_ ± 0.001_se_)	0–0.012 (0.005_mean_ ± 0.0003_se_)	0.005–0.029 (0.014_mean_ ± 0.001_se_)	0–0.014 (0.007_mean_ ± 0.0003_se_)	*tigA*
	7b	0.003–0.050 (0.037_mean_ ± 0.002_se_)	0.004–0.029 (0.015_mean_ ± 0.001_se_)	0.008–0.032 (0.019_mean_ ± 0.001_se_)	0.005–0.023 (0.015_mean_ ± 0.001_se_)	All
	7c	0.058–0.060 (0.059_mean_ ± 0.001_se_)	0.016–0.022 (0.020_mean_ ± 0.002_se_)	0.024–0.029 (0.027_mean_ ± 0.001_se_)	0.020–0.024 (0.022_mean_ ± 0.001_se_)	All
	7d	0.018	0.004	0.004	0.003	All
	**Overall**	**0–0.085 (0.050**_mean_ ±**0.001**_se_**)**	**0–0.112 (0.040**_mean_ ±**0.002**_se_**)**	**0.004–0.062 (0.032**_mean_ ±**0.001**_se_**)**	**0–0.041 (0.023**_mean_ ±**0.001**_se_**)**	***tigA***
8	8a	0–0.067 (0.041_mean_ ± 0.003_se_)	0–0.038 (0.018_mean_ ± 0.002_se_)	0.001–0.033 (0.019_mean_ ± 0.002_se_)	0–0.033 (0.019_mean_ ± 0.001_se_)	*tigA*^a^
	8b	0.007–0.065 (0.055_mean_ ± 0.003_se_)	0–0.028 (0.017_mean_ ± 0.002_se_)	0.003–0.073 (0.052_mean_ ± 0.003_se_)	0–0.060 (0.040_mean_ ± 0.004_se_)	*cox1* and *tigA*
	8c	0.046–0.070 (0.061_mean_ ± 0.004_se_)	0.014–0.074 (0.044_mean_ ± 0.008_se_)	0.040–0.072 (0.054_mean_ ± 0.005_se_)	0.023–0.047 (0.035_mean_ ± 0.004_se_)	All
	8d	0.034–0.040 (0.038_mean_ ± 0.002_se_)	0.027–0.040 (0.034_mean_ ± 0.004_se_)	0.047–0.063 (0.057_mean_ ± 0.005_se_)	0.028–0.038 (0.033_mean_ ± 0.003_se_)	All
	**Overall**	**0–0.104 (0.070**_mean_ ±**0.001**_se_**)**	**0–0.136 (0.069**_mean_ ±**0.002**_se_**)**	**0.001–0.133 (0.079**_mean_ ±**0.002**_se_**)**	**0–0.113 (0.057**_mean_ ±**0.001**_se_**)**	***tigA***^a^
9	9a1	0.001–0.091 (0.034_mean_ ± 0.003_se_)	0.005–0.090 (0.047_mean_ ± 0.003_se_)	0–0.067 (0.031_mean_ ± 0.002_se_)	0–0.052 (0.019_mean_ ± 0.002_se_)	ITS
	9a2	0.003–0.013 (0.009_mean_ ± 0.003_se_)	0.001–0.013 (0.009_mean_ ± 0.004_se_)	0.002–0.004 (0.003_mean_ ± 0.001_se_)	0–0.001 (0.001_mean_ ± 0.0003_se_)	*cox1* and *tigA*
	9a3	0.051	0.006–0.073 (0.050_mean_ ± 0.022_se_)	0.038	0.001–0.021 (0.014_mean_ ± 0.006_se_)	*cox1*, ITS, and *tigA*
	9b	0.046–0.074 (0.063_mean_ ± 0.008_se_)	0.010–0.068 (0.048_mean_ ± 0.019_se_)	0.016–0.064 (0.048_mean_ ± 0.016_se_)	0.019–0.043 (0.032_mean_ ± 0.007_se_)	All
	**Overall**	**0.001–0.112 (0.057**_mean_ ±**0.002**_se_**)**	**0.001–0.188 (0.110**_mean_ ±**0.004**_se_**)**	**0–0.075 (0.046**_mean_ ±**0.001**_se_**)**	**0–0.065 (0.034**_mean_ ±**0.001**_se_**)**	**ITS and** ***cox1***^a^
10		**0.032–0.088 (0.064**_mean_ ±**0.003**_se_**)**	**0.014–0.142 (0.096**_mean_ ±**0.010**_se_**)**	**0.037–0.111 (0.087**_mean_ ±**0.005**_se_**)**	**0.020–0.077 (0.056**_mean_ ±**0.004**_se_**)**	**All**

*(Sub)clades containing species with identical sequences are highlighted in orange. (Sub)clades containing almost identical sequences (distance ≤ 0.001) are highlighted in yellow. Overall species distance and recommended marker(s) for each clade are in bold*.

a *Taxa with almost identical sequences (distance ≤ 0.001) for the respective markers were found*.

Among the four markers, *tigA* and *cox1* had relatively high distances within most (sub)clades. Species with identical sequences were found in 3 clades and 2 subclades for *cox1*, 2 clades and 2 subclades for *tigA*, 5 clades, and 8 subclades for β*-tub*, and 6 clades and 5 subclades for ITS (Table 3). Species pairs with identical sequences of each marker in individual (sub)clades are listed in Table 4.

**Table 4:** Species pairs with identical sequences for four genetic markers.

**(Sub)clade**	**Spp. pairs**		**ITS**	***tigA***	***β-tub***	***cox1***
1c	*P. andina*	*P. infestans*	x	x		
	*P. andina*	*P. mirabilis*	x			
	*P. infestans*	*P. mirabilis*	x			
1	*P. iranica*	*P. infestans*				x
2a	*P. occultans*	*P. terminalis*			x	
2b	*P. capsici*	*P. mexicana*			x	
5	*P. agathidicida*	*P. castaneae*	x			
6a	*P. kwongonina*	*P. rosacearum*			x	
6b	*P. chlamydospora*	*P. gonapodyides*	x			
7a	*P. alni*	*P. × incrassata*	x			
	*P. alni*	*P. × multiformis*	x		x	x
	*P. europaea*	*P. flexuosa*	x			
	*P. fragariae*	*P. rubi*	x			
	*P. uniformis*	*P. alni*	x			
	*P. uniformis*	*P. × incrassata*	x			
	*P. uniformis*	*P. × multiformis*	x			
	*P. × incrassata*	*P. ×* multiformis	x			
8a	*P. cryptogea*	*P. erythroseptica*	x		x	x
8b	*P. lactucae*	*P. pseudolactucae*	x			
	*P. primulae*	*P. taxon parsley*	x		x	
9a1	*P. hydropathica*	*P. parsiana*			x	
	*P. hydropathica*	*P. virginiana*			x	
	*P. hydropathica*	*P*. aff. parsiana G1		x	x	
	*P. hydropathica*	*P*. aff. parsiana G2			x	
	*P. hydropathica*	*P*. aff. parsiana G3			x	
	*P. parsiana*	*P. virginiana*			x	
	*P. parsiana*	*P*. aff. parsiana G1			x	
	*P. parsiana*	*P*. aff. parsiana G2			x	
	*P. parsiana*	*P*. aff. parsiana G3			x	
	*P. virginiana*	*P*. aff. parsiana G1			x	
	*P. virginiana*	*P*. aff. parsiana G2			x	
	*P. virginiana*	*P*. aff. parsiana G3			x	
	*P*. aff. parsiana G1	*P*. aff. parsiana G2			x	
	*P*. aff. parsiana G1	*P*. aff. parsiana G3			x	
	*P*. aff. parsiana G2	*P*. aff. parsiana G3			x	
9a2	*P. macrochlamydospora*-G2	*P. quininea*			x	

For clade 1, there were no identical β*-tub* sequences, while identical species pairs were found for the other three markers (Table 3). No identical sequences of *cox1* or *tigA* were found in clades 2 and 6. All markers except for the ITS had acceptable to high (minimum distance = 0.002–0.027) resolution within clade 3, 4, and 5. Identical ITS sequences were found in clade 5. Almost identical ITS sequences (distance ≤ 0.001) were found in clades 3 and 4. *tigA* was the only marker of unambiguity for clade 8, although almost identical *tigA* sequences were present in that clade (Table 3). No identical sequences of *cox1* or ITS were found in clade 9. All markers provided high resolution among clade-10 species.

### Comparison of individual-marker trees with concatenated-sequence tree

The resulted clade assignments and clade affiliation of individual species (Table 1) based on the concatenated-sequence tree (TreeBASE S22998) were nearly identical to those generated in previous phylogenetic studies (Blair et al., 2008; Martin et al., 2014; Yang et al., 2017) except that the placement of *P. quercina* was ambiguous.

All trees from sequences of the three nuclear markers had similar topologies (score = 75.3–81.7%) to those of the concatenated-sequences trees in both ML and NJ analyses. In contrast, *cox1* sequences produced trees of distinct topologies (TreeBASE S22998). The overall topological similarities to the concatenated-sequences trees were approximately 45% lower than those of nuclear markers in both analyses (Table 5).

**Table 5:** Similarity of individual-marker trees to concatenated-sequence tree.

**Marker**	**Overall topological score**^**a**^	

	**Maximum likelihood**	**Neighbor joining**
*cox1*	36.6	37.1
ITS	81.0	77.9
*tigA*	78.4	81.7
*β-tub*	75.3	79.2

a *Scores were calculated using Compare2Trees*.

## Discussion

This study identified four most informative genetic markers for identifying *Phytophthora* species: *cox1*, ITS, *tigA*, and β*-tub*. The resolution of each marker depended on (sub)clade. These results along with the signature sequences generated by Cooke et al. (2000), Kroon et al. (2004), Blair et al. (2008), Martin et al. (2014), and Yang et al. (2017) enable first responders, diagnosticians, and researchers to identify *Phytophthora* isolates with confidence at minimal cost in the briefest time possible.

### ITS

Using the ITS sequence to identify *Phytophthora* isolates has several advantages. First, the ITS region has the most comprehensive sequence database when compared to other markers. As this marker has been proposed as the barcode for fungi and oomycetes (Seifert, 2009) and later designated as the barcode for all fungi (Schoch et al., 2012), almost all known *Phytophthora* taxa have been sequenced for the ITS region. Subsequently, sequencing the ITS region of unknown *Phytophthora* isolates has become a common practice in research labs and plant disease clinics. Second, the ITS region amplified by the primer pair ITS6/ITS4 has the best universality across the genus and the highest PCR consistency among markers evaluated in this study (Table 1). Third, the clade affiliations of individual species based on the ITS sequences mostly accord with those based on multilocus sequence data (Table 5).

Despite the above merits, ITS alone is not sufficient to identify all *Phytophthora* isolates to the species level. Identical ITS sequences have been observed in 16 pairs of species in clades 1, and 5–8, more than any of *tigA*, β*-tub*, and *cox1* (Table 4). These identical and other almost identical ITS sequences (distance ≤ 0.001 or difference between sequences ≤ 10 bases) were found in clades 1–9, while those for *tigA*, β*-tub*, and *cox1* only occurred in 4, 5, and 4 clades, respectively (Table 3). This result indicates that it is important to use additional markers to identify *Phytophthora* isolates in all clades, perhaps with the exception of clade 10.

Due to its high universality, availability, and PCR consistency, the ITS region is an ideal first genetic marker for identifying *Phytophthora* isolates to clade.

### *cox1* amplified by the primer pair COXF4N/COXR4N

The *cox1* has the highest genus-wide resolution among the evaluated markers (Table 2). Only three species pairs with identical *cox1* sequences were found (Table 4). However, using *cox1* alone for identifying *Phytophthora* isolates presents a few problems. First, *cox1* had the second lowest PCR success rate (Table 2). In cases, adjusting MgCl_2_ and BSA concentrations, and annealing temperature were required for a successful amplification. However, it is important to note that the presented PCR success rates (Table 2) were calculated based on all PCR amplifications done by the two authors in the past 6 years, while many other factors could influence the PCR success rate, such as the quality of DNA templates and primers, and different PCR operators and thermocyclers. Second, (sub)clade-classification solely by *cox1* may conflict with those assigned by multi-locus analyses (Table 5). Thus, using *cox1* alone may lead to misidentification of unknown *Phytophthora* isolates at the (sub)clade-level. Third, due to the uniparental inheritance of mitochondria, it is impossible to separate a hybrid *Phytophthora* species from its maternal parent based on the *cox1* sequence. This is increasingly important as *Phytophthora* hybrids have been commonly found in many ecosystems (Nirenberg et al., 2009; Man in't Veld et al., 2012; Nagel et al., 2013; Yang et al., 2014; Husson et al., 2015; Jung et al., 2017). This problem not only occurs for *cox1*, but also for other mitochondrial markers that were not evaluated in this study.

### tigA

The *tigA* has moderately high genus-wide resolution (Table 1). High similarity in topology between the *tigA* tree and the multi-locus tree (Table 5) makes this marker useful in assigning *Phytophthora* isolates to (sub)clades. Additionally, it had excellent resolution within most individual (sub)clades. Species with identical *tigA* sequences were found only in subclades 1c and 9a1 (Table 3). However, this marker has the lowest PCR success rate of 71%. In addition, internal primers were usually required for sequencing (Table 1), which increases the cost. Both factors potentially compromise the usefulness of the *tigA* marker.

### β-tub

The marker β*-tub* had the fourth highest genus-wide resolution. High similarity in topology between the β*-tub* tree and the multi-locus tree (Table 5) makes this marker also useful for assigning *Phytophthora* taxa to (sub)clades. Like ITS, β*-tub* is easy to amplify (Table 2), which further adds to its usefulness. However, 22 species pairs in clades 2, and 6–9 have identical β*-tub* sequences (Table 3). Thus, β*-tub* does not have the resolution required for identifying *Phytophthora* isolates to species in these clades.

## Conclusions

Among the nine genetic markers evaluated in this study, *cox1*, ITS, *tigA*, and β*-tub* were the most informative for the genus *Phytophthora*. Both ITS and β*-tub* were easy to amplify but had limited species distance within some (sub)clades. Comparatively, *cox1* and *tigA* had high resolution within most (sub)clades but they were relatively difficult to amplify. In addition, *cox1* was not useful for assigning species to (sub)clades nor for identifying hybrid taxa. Taken together, a two-step approach is recommended: identifying unknown *Phytophthora* isolates to clade level with ITS sequences then to species level with one or more additional markers (Table 3). For example, β*-tub* can be used to readily identify all species in clade 1, *cox1* for clade 2, and *tigA* for clades 7 and 8 (Table 3).These recommendations along with available signature sequences enable first responders, diagnosticians, and researchers to identify *Phytophthora* isolates with confidence at reduced time and cost.

## Author contributions

XY and CH conceived and designed the experiments, contributed reagents, materials, analysis tools, and wrote the paper. XY performed the experiments and analyzed the data.

### Conflict of interest statement

The authors declare that the research was conducted in the absence of any commercial or financial relationships that could be construed as a potential conflict of interest.
